# Limited evolutionary rescue of locally adapted populations facing climate change

**DOI:** 10.1098/rstb.2012.0083

**Published:** 2013-01-19

**Authors:** Katja Schiffers, Elizabeth C. Bourne, Sébastien Lavergne, Wilfried Thuiller, Justin M. J. Travis

**Affiliations:** 1 Laboratoire d'Ecologie Alpine, Université Joseph Fourier, Grenoble 1, UMR-CNRS 5553, BP 53, 38041 Grenoble Cedex 9, France; 2 Institute of Biological and Environmental Sciences, University of Aberdeen, Zoology Building, Tillydrone Avenue, Aberdeen AB24 2TZ, UK; 3 The James Hutton Institute, Craigiebuckler, Aberdeen AB15 8QH, UK; 4 Institut für Biologie—Botanik, Freie Universität Berlin, Altensteinstrasse 6, 14195 Berlin, Germany

**Keywords:** allelic model, dispersal, gene flow, habitat heterogeneity, migration load, rapid adaptation

## Abstract

Dispersal is a key determinant of a population's evolutionary potential. It facilitates the propagation of beneficial alleles throughout the distributional range of spatially outspread populations and increases the speed of adaptation. However, when habitat is heterogeneous and individuals are locally adapted, dispersal may, at the same time, reduce fitness through increasing maladaptation. Here, we use a spatially explicit, allelic simulation model to quantify how these equivocal effects of dispersal affect a population's evolutionary response to changing climate. Individuals carry a diploid set of chromosomes, with alleles coding for adaptation to non-climatic environmental conditions and climatic conditions, respectively. Our model results demonstrate that the interplay between gene flow and habitat heterogeneity may decrease effective dispersal and population size to such an extent that substantially reduces the likelihood of evolutionary rescue. Importantly, even when evolutionary rescue saves a population from extinction, its spatial range following climate change may be strongly narrowed, that is, the rescue is only partial. These findings emphasize that neglecting the impact of non-climatic, local adaptation might lead to a considerable overestimation of a population's evolvability under rapid environmental change.

## 1. Introduction

Facing one of the most drastic global changes in the Earth's history, a fundamental objective of current ecological and evolutionary research is to understand and predict species’ responses to changing environmental conditions [1]. Three key types of response may ameliorate the threat of extinction: buffering against negative effects of deteriorating habitat by phenotypic plasticity [2–5], tracking suitable climate through range shifting [6,7] and adapting to changing conditions by rapid evolution [8,9]. Some authors suggest that most species will more likely shift their distributional ranges or respond by phenotypic plasticity rather than adapt *in situ* to new conditions [6,10]. This is mainly because plasticity and range shifting may be substantially faster in matching phenotypic preferences with environmental conditions than evolutionary processes. Nonetheless, a number of species have been shown to adapt with remarkable rapidity in response to environmental change [11,12], and numerous studies have identified heritable population differentiation in ecologically relevant traits, providing indirect evidence for the potential of adaptive evolution over ecological time-scales [8,13,14]. It thus seems imperative to consider the role of evolutionary rescue—the phenomenon of once declining populations evolving back to positive growth by evolutionary adaptation—in assessments of the likely impacts of global change on species abundance, distribution and persistence.

The theoretical foundations of adaptive dynamics have been established over the past decades by a growing number of studies in the fields of population and quantitative genetics. A key theorem states that the rate of adaptation is predominantly driven by the amount of available additive genetic variance and the strength of environmental selection [15,16]. In principle, given sufficient genetic variance, populations should adapt to virtually any environmental condition [17]. However, under natural conditions, an often-complex interaction between demographic processes and evolutionary dynamics may result in failure of adaptation and ultimate extinction of the population [18–20]. To gain initial insights into such interactive processes, synthetic approaches linking genetics with population demography are being applied increasingly frequently, addressing questions on, e.g. the formation of species range edges [21–23] and invasion dynamics [24,25], including invasion dynamics in heterogeneous landscapes [26,27].

In the context of eco-evolutionary processes, dispersal is a key determinant of population dynamics, owing to its impact on both spatial demography [28,29] and the speed of local adaptation [29–31]. As a consequence, dispersal is likely to be crucial for evolutionary rescue. The main responsible mechanism is the spreading of newly arising, beneficial alleles throughout a population's distributional range [32–35]. In a recent study, Bell & Gonzalez [35] empirically tested these theoretical predictions with an experiment on bakers yeast: they demonstrated that spatially structured populations had a significantly higher chance of surviving a period of deteriorating growth conditions and adapting to the new state, when dispersal allowed for gene flow across subpopulations.

In contrast to its beneficial effect for rapid adaptation under temporally changing conditions, dispersal is known to have an overall negative influence on population fitness under most scenarios of local adaptation [36,37]. In a spatially heterogeneous environment, mismatches between immigrants' genotypes and the environmental conditions at their destination locations result in a reduction in overall fitness, termed migration load. With analytical predictions and individual-based simulations, Lopez *et al.* [37] have illustrated how, under gene flow through both pollen and seed movement, migration load increases with the degree of habitat heterogeneity. In a further theoretical study, Alleaume-Benharira *et al*. [38] demonstrated that in a patchy population, distributed across an environmental gradient, intermediate rates of dispersal optimized fitness. This was the result of a trade-off between some dispersal having benefits in terms of purging deleterious alleles, especially from smaller marginal populations, and increasing dispersal resulting in higher migration load owing to gene flow between patches of differing local conditions.

Clearly, adaptation to heterogeneous habitat and temporal changes in environmental conditions often occur hand in hand [39], confronting populations with multiple sources of potential maladaptation. However, the few existing studies investigating population responses to a spatially and temporally changing optimum focus predominantly on a single environmental variable [40–42]. Such an approach neglects a situation that is likely to be very common in natural conditions, that is where the spatially heterogeneous conditions driving local adaptation of populations differ from those that undergo temporal changes. A simple example can illustrate this statement. Many plant populations are locally adapted to varying abiotic conditions (e.g. edaphic factors) or biotic context (e.g. presence/absence of herbivores), but the mosaic of this local adaptation will mostly be decoupled from currently changing climatic gradients. Under these circumstances, the central question is: how do the contradictory effects of dispersal influence the evolutionary response of populations to environmental change?

In this study, we address the above question by integrating the key processes that have until now typically been studied separately: the role of dispersal as the mechanism distributing adapted alleles across populations and the feedbacks between dispersal and local adaptation. We do this within the context of an allelic model, where population genetics are coupled with population ecology by conditioning demographic rates on the match of genetically variable traits to environmental characteristics. We use our model to examine how the interplay between dispersal and local adaptation across spatially heterogeneous habitats influences the probability of evolutionary rescue of populations facing changing climatic conditions. We also examine how the genetic architecture of adaptive traits modulates this interplay.

## 2. The model

We developed an allelic, spatially explicit and individual-based simulation model to investigate the interactive effects of gene flow and local adaptation on the evolutionary response of populations to environmental change. The full source code and an accompanying readme file are available as electronic supplementary material, and a maintained version of the model is downloadable from http://www.katja-schiffers.eu/docs/allele_model.zip.

The model organism we had in mind during implementation was a bisexual, annual plant species with xenogamous breeding system. Population dynamics take place within a continuous region of 32 × 32 grid cells. To avoid arbitrary edge effects, the area is simulated as a torus, i.e. the edges of both axes are joined. Grid cells are characterized by two environmental conditions: (i) local environmental conditions such as edaphic parameters or particular biotic settings, which follow a fractal distribution and are stable over time; and (ii) climatic conditions, e.g. maximal annual temperature, which change during the simulated period. For simplicity, we assume climate to be homogeneous across space. Each grid cell can support a number of individuals, the maximal number given by the local carrying capacity, which is constant across the region. Individuals are diploid, carrying two copies of either one or several chromosomes coding for an individual's level of adaptation to climatic and local environmental conditions. Individuals are located in continuous space and are assigned to the grid cell within which their *x-* and *y*-coordinates fall.

Within each generation, the following processes are simulated: (i) reproduction with mutation, recombination, gamete dispersal and subsequent death of the parental generation, (ii) dispersal of the offspring, (iii) selection acting on the survival probabilities of the juveniles, and (iv) density-dependent mortality. Selection takes the form of density-independent, hard selection for an individual's adaptation to both climatic and local environmental conditions.

### (a) Genetic architecture

A number of previous studies have shown that traits affecting species' adaptation, particularly to climatic conditions, are usually polygenic. For example, 12 quantitative trait loci have been identified for climatic adaptation in *Arabidopsis thaliana* [43], 33 for bud-flush, nine for autumn cold hardiness and nine for spring cold hardiness in *Pseudotsuga menziesii* [44] (see also Falconer & Mackay [45] for a general overview). On the basis of this information, we simulated genomes composed of *n* = 15 loci for each of the two considered traits. To represent two contrasting scenarios of linkage, we considered the genome to be composed either of one or several pairs of chromosomes. In the first case, all loci are situated on a single chromosome and, as we do not allow for crossovers during recombination, the loci are fully linked. Effectively, this could also be considered a single locus with multiple alleles and pleiotropic effects. In the second case, we assume the opposite possible extreme case of no linkage. This may correspond to a situation where a genome is made of 30 chromosome pairs each carrying a single locus, implying completely independent inheritance of alleles. Or, this might mimic a situation where all the loci are on a single (or multiple) chromosome, but with sufficient distance between the loci and sufficient frequency of crossover events that they are effectively unlinked. Alleles are described by continuous values and are additive within and between loci, i.e. neither epistatic nor pleiotropic effects are considered. Individuals' phenotypes are directly determined by their genotypes, that is, environmental effects on phenotypes are neglected, and heritability is thus assumed to be unity [21–23,46].

### (b) Simulated processes

#### (i) Reproduction

All individuals can potentially bear offspring. The number of ovules produced by each individual is drawn from a Poisson distribution with average *R* = 100. Whether, and by which mating partner, single ovules are fertilized is modelled stochastically with the probabilities of fertilization derived from the individuals' distances and the shape of the pollen dispersal kernel (see §2*b*(ii)). Gametes are composed by duplicating parental chromosomes, one of each homologous pair being chosen randomly. Alleles mutate with a probability of *μ* = 10^−7^, which represents the average rate found for the annual plant species *Arabidopsis thaliana* [47–49]. The mutational effect, i.e. the amount by which the allelic value is changed, is drawn from a zero-mean normal distribution with variance *α*^2^ = 0.2, approximately fitting empirical observations [47].

#### (ii) Dispersal

There are two phases of dispersal in each generation cycle: pollen dispersal and offspring dispersal. Both are characterized by lognormal, isotropic dispersal kernels with an average distance *d* for both gametes and offspring and a shape parameter of 0.5. The lognormal distribution has been found to adequately represent both local and long-distance dispersal [50].

Offspring dispersal is simulated explicitly: dispersal distance and direction are chosen randomly with probabilities following the shape of the dispersal kernel. The offspring is positioned at the resulting *x*/*y*-coordinates, respecting torroidal boundary conditions.

To gain sufficient computational efficiency, we do not explicitly simulate the dispersal of pollen. Instead, we use the following algorithm. As for offspring dispersal, *x*/*y*-coordinates are chosen randomly in the neighbourhood of the focal individual. The mating partner is then randomly drawn from all individuals inhabiting the grid cell within which the random position is located. In case the selected grid cell is empty, the procedure is repeated up to 99 times. If all trials are unsuccessful, we assume the ovule not to be fertilized.

To test for potential undesirable effects of this simplification, we also developed implementation of gamete dispersal that is more precise in the sense of linking fertilization probability to the exact distance between individuals. For each individual of the population, the probability of fertilizing a specific ovule is calculated based on the inter-individual distances and the shape of the dispersal kernel. Following that, the probability of no fertilization can be determined. Rescaling all resulting probabilities so that they add up to unity then allows sampling of the pollen donor by a draw of a uniform random number between zero and unity. Comparisons between the two approaches showed that there are no obvious differences at the level of evolutionary or demographic dynamics. We thus chose the former, computationally much less intensive method.

#### (iii) Selection

Selection acts on population demography by modulating juvenile survival probability. Each individual's survival probability *W* is calculated as the product of its condition related to climate *W*_C_ and its condition related to the local environment *W*_E_. Both of these values, *W*_C,E_, are functions of the difference between the individual's phenotype *z*_C,E_, and the optimal phenotype under the current climatic or local environmental conditions *Θ*_C,E_. They follow a normal distribution with maximum unity and variance **ω*^2^*_C,E_:
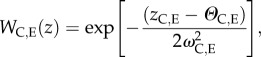
where **ω*^2^* is traditionally referred to as selection strength, but can also be interpreted as a measure of the species’ tolerance to suboptimal conditions, i.e. its niche breadth [40]. Here, for the default parameter settings, this value was fixed at 0.1, resulting in *W*_C_ < 0.01 and thus in a negative population growth rate (given the average number of offspring per individual is 100) when temperature has changed by approximately 1°C, assuming the phenotype is fixed. In a number of additional runs, selection strength was reduced by increasing **ω*^2^* to 0.2.

#### (iv) Density-dependent mortality

We assume a simple ceiling form of density dependence (similar to [39,51]): whenever the number of individuals within a grid cell exceeds its carrying capacity, *K*, resident individuals are subjected to a density-dependent mortality with probability of survival = 1−*K*/*N*.

### (c) Simulations

Simulations were run to test the interactive effects of dispersal, habitat heterogeneity and linkage on population dynamics and the likelihood of survival under climate change (see table 1 for model parameters). The average dispersal distance *d* was set to 0.05 grid cell lengths for the first set of simulations and then repeatedly doubled up to a distance of 6.4. Habitat heterogeneity *h*_H_ was controlled by modifying the range of possible local environmental conditions from 0 units, i.e. no heterogeneity, to a maximum of six units in steps of 1. For testing the effect of linkage, the two contrasting scenarios of complete versus no linkage were compared. The remaining model parameters were kept constant across simulations. For all possible 112 combinations of *d*, *h*_H_ and linkage, we ran 100 replicates, recording population size over time and the population average and variance of individuals' survival probabilities, *W*_C,E_, as a measure for their conditions.

**Table 1.** Parameter values for simulation runs.

parameter	description	values
*V*	rate of climate shift	two units per 100 years
*H*	Hurst exponent	0.2
*h*_H_	habitat heterogeneity	0, 1, 2, 3, 4, 5, 6
*K*	carrying capacity per grid cell	5
*R*	mean number of offspring	100
*D*	mean dispersal distance	0.05, 0.1, 0.2, 0.4, 0.8, 1.6, 3.2, 6.4
*d*_shape_	shape factor dispersal kernel	0.5
*L*	linkage between loci	fully linked, free recombination
*M*	mutation rate per locus	10^−7^
*α*^2^	variance of mutational effect	0.2
**ω*^2^*	selection strength	0.1, 0.2

Landscapes were initialized with a value of 25°C for the climatic conditions and a Hurst exponent of 0.2 for the fractal distribution of local environmental conditions, their amplitude being controlled by *h*_H_. The values assigned to *h*_H_ resulted in average differences between neighbouring cells of 0.03, 0.09, 0.15, 0.38, 0.9 and 1.46 units, respectively. The spatial population was initialized by colonizing each grid cell with three individuals. Individuals were, on average, optimally adapted to both climatic and local environmental conditions, but exhibited normally distributed additive genetic variation with a within-cell variance of 0.01. This value corresponds approximately to the mutation–selection equilibrium reached after 1000 generations in previous test runs under stable conditions (see the electronic supplementary material, figures S1–S4). It has to be noted that allelic values typically did not follow normal distributions at the end of these runs, particularly when habitat heterogeneity was low. However, comparisons with additional simulations with no initial genetic variation showed that resulting population parameters were not influenced by the chosen starting conditions (see the electronic supplementary material, figure S5). For the main analysis, climate change was simulated by keeping the temperature constant over the first 200 generations and then gradually increasing it by 2.0°C over the following 100 time-steps. After this period of change, the new climatic conditions were assumed to be stable until the end of 500 simulation years.

## 3. Results

In test runs without environmental change, population size, average individual fitness and additive genetic variance were stable over time, unless mean dispersal distances were too small to ensure a sufficient number of fertilized ovules to keep growth rates higher than unity. When introducing a shift in climate, population size started to decline at the point where the average individual phenotype lagged so far behind the optimum *Θ* that *W* < 1/*R*. In simulations where the mutation rate *μ* was set to zero, populations inevitably died, because standing genetic variation alone did not provide enough scope for full adaptation to new conditions. With the default value for *μ* = 10^−7^, an average family size of 100 and a carrying capacity around 5000 individuals, mutations occurred on average once per generation and locus. In combination with the given variance of the mutational effect (*α*^2^) = 0.2 and a selection strength (*ω*^2^) = 0.1, allelic dynamics resulted in a slow disruption of the initial normal distribution of allelic values (see the electronic supplementary material, figures S1–S4) during periods of stable climate. During phases of temperature rise, mainly the fixation of rare, large mutations contributed to the adaptation process to the new conditions (results not shown), leading to punctuated phases of rapid evolution as, for example, described in Holt *et al*. [39].

**Figure 1.**
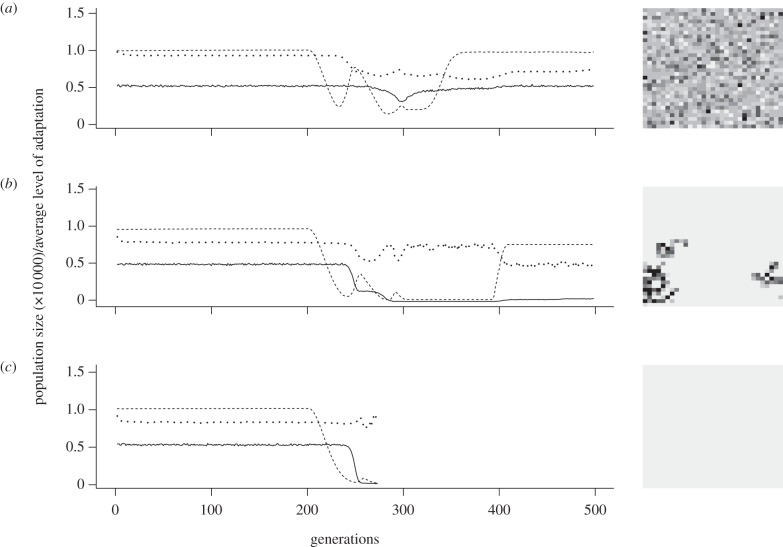
Three example runs depicting (*a*) full rescue, (*b*) partial rescue, and (*c*) population extinction. Solid lines represent population size, short dashed lines represent the level of adaptation to climatic conditions, *W*_C_, and long dashed lines represent the level of adaption, *W*_E_, to local environmental conditions. On the right-hand side the density of individuals is shown after 500 time-steps with darker values indicating higher densities.

Population responses to rapid climate change fitted into three general classes, depending upon the values of some key model parameters. We first describe the three main categories of response (figure 1), before providing some detail on how the key parameters influenced the outcome.

*Complete evolutionary rescue* occurred when there was a sufficient number of beneficial mutations and when they were able to spread unhampered across the landscape. This class of response was typically characterized by an initial phase during which, as the climate began to change, individuals' survival probabilities declined. Subsequently, as one or more beneficial mutations occurred and spread across the landscape, the average individual's fitness increased, the total population size fully recovered and ultimately individuals' phenotypes were a good match to the new climate conditions (figure 1*a*).

*Partial evolutionary rescue* occurred under conditions where beneficial alleles arose but were unable to spread owing to ineffective gene flow across space. In this class of response, only fragments of what was previously fully occupied habitat were populated following climate change. This effective reduction in the suitable habitat niche for the population sometimes resulted in substantially reduced total population sizes following climate change (figure 1*b*). Importantly, this effect was persistent, lasting until the end of simulations, which ran for 200 generations after climate change ceased.

*Extinction*, due to the failure of evolutionary rescue, occurred when the frequency of beneficial mutations was too low. Under these conditions, individuals' phenotypes rapidly became very poorly matched to the prevalent climatic conditions, resulting in lower offspring viability and ultimately a non-viable population (figure 1*c*).

### (a) Effects of dispersal and habitat heterogeneity

**Figure 2.**
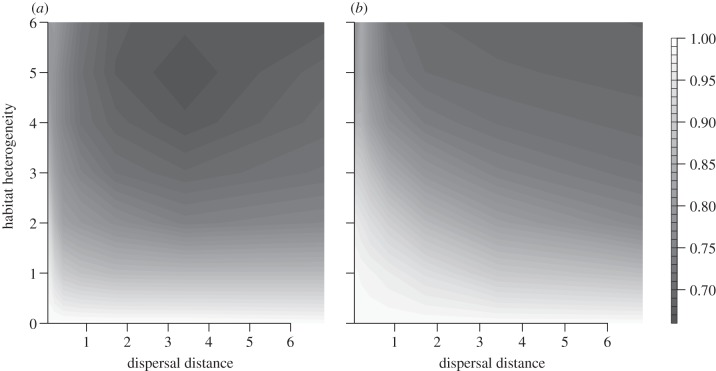
Average values for the level of adaptation to local environmental conditions, *W*_E_, during the phase of temperature rise for (*a*) full linkage and (*b*) free recombination of loci. Depicted are the average values over 100 replicates for all combinations of habitat heterogeneity *h*_H_ and average dispersal distances *d* in grid cell length.

In accordance with our expectations based on previous studies [36,37], in a spatially heterogeneous environment, dispersal generally had a negative effect on individuals' levels of adaptation to environmental conditions *W*_E_ (figure 2).

In scenarios of full linkage, the level of adaptation increased again for very high values of dispersal and heterogeneity (figure 2*a*), owing to an increased mortality of strongly maladapted individuals and consequently higher averages for the surviving fraction of the population (results not shown).

**Figure 3.**
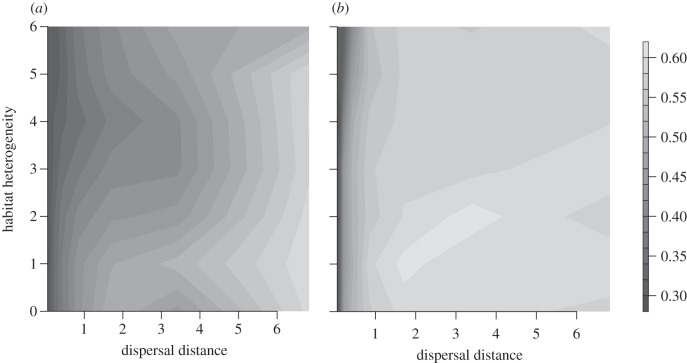
Average values for the level of adaptation to climatic conditions, *W*_C_, during the phase of temperature rise for (*a*) full linkage and (*b*) free recombination of loci. Depicted are the average values over 100 replicates for all combinations of habitat heterogeneity *h*_H_ and average dispersal distances *d* in grid cell length.

On the other hand, model results also confirmed the beneficial effect of dispersal on a population's adaptation to temporally changing conditions. This was demonstrated by increasing values of *W*_C_ with increasing dispersal distances (figure 3). However, this pattern appeared to be more sensitive to stochastic effects than results regarding the adaptation to local environmental conditions.

**Figure 4.**
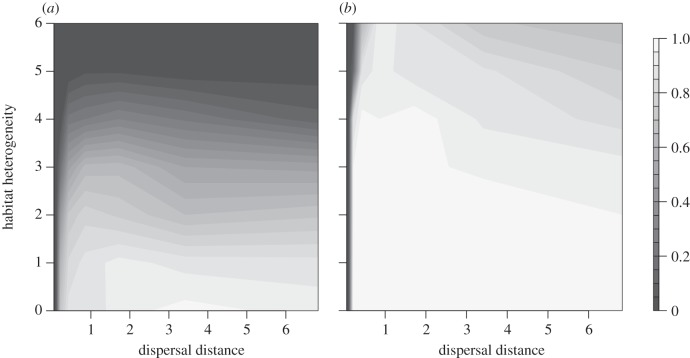
Probability of full rescue depending on habitat heterogeneity *h*_H_ and average dispersal distances *d* in grid cell length for (*a*) full linkage and (*b*) free recombination of loci. Calculated from 100 simulation runs for each parameter combination.

**Figure 5.**
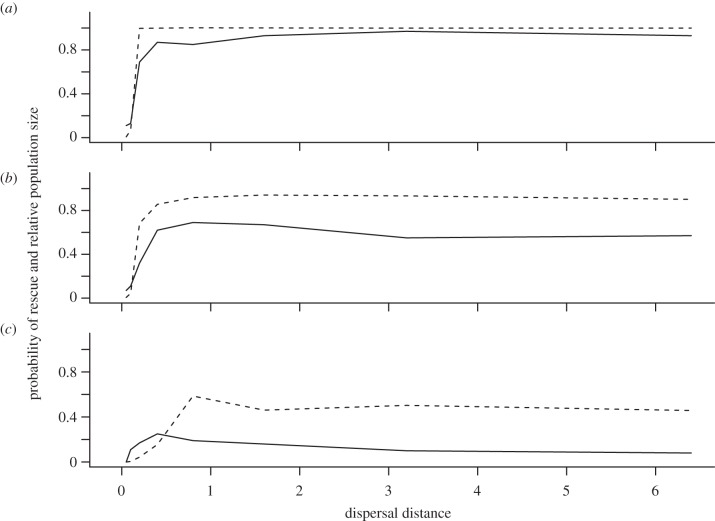
Probability of evolutionary rescue (partial and full rescue, solid lines) and relative population sizes (population size at generation 500/*K*, extinctions excluded, dashed lines) in dependence on dispersal distance *d* for habitat heterogeneities of (*a*) *h*_H_ = 0, (*b*) *h*_H_ = 3, and (*c*) *h*_H_ = 5. Results are based on 100 simulation runs for full linkage of loci.

The likelihood of evolutionary rescue was strongly reduced or even hindered for a range of dispersal distances, for which rapid adaptation would have been possible without local adaptation (figure 4). Because both high dispersal distances, as well as very low distances, decreased the probability of evolutionary rescue, highest survival rates were observed for intermediate values between 0.4 and 2 grid cell units. Within that range, the peak of rescue probability depended on the level of habitat heterogeneity and shifted towards shorter dispersal distances with increasing heterogeneity (figure 5*a–c*).

With increasing spatial heterogeneity, there was also an increased likelihood that, when rescue occurred, it was only partial. Thus, while the population had at least some probability of surviving climate change through evolutionary rescue, the landscape was not fully occupied after climate change and the total population size was substantially reduced (figure 5). Under a heterogeneity of *h*_H_ = 5, the average relative population size (of the surviving populations) at the end of the simulation time was, across a broad range of dispersal distances, reduced to an average of around 50 per cent of pre-climate-change densities (figure 5*c*). Interestingly, the parameter values that maximized the probability of rescue did not necessarily result in a more complete rescue. For example, when *h*_H_ = 5, there was the greatest probability of population survival when dispersal = 0.4. For this scale of dispersal, however, surviving populations were reduced on average to roughly one-sixth of their initial abundance. By contrast, when dispersal occurred across a greater range (e.g. dispersal = 2.5), the populations survived only 10 per cent of the time, but then recovered to an average 50 per cent of initial abundance.

### (b) Effect of linkage

The assumptions regarding the form of linkage had a strong effect on the overall probability of evolutionary rescue. Independent inheritance allowed for much faster adaptation to both spatially (figure 2) and temporally changing conditions (figure 3) so that the negative effect of local adaptation was strongly ameliorated (figure 4). However, the overall pattern of intermediate dispersal distances resulting in highest evolutionary potential was consistently observed for both scenarios.

## 4. Discussion

Global environmental change is confronting natural populations simultaneously with rapid climate change and increasing habitat loss and deterioration. The combination of habitat fragmentation and limited dispersal will prevent many populations from tracking suitable climate in space. For these species, *in situ* adaptation to changing climate is likely to provide the only natural means of avoiding ultimate extinction. Understanding the factors determining the likelihood that populations adapt sufficiently rapidly to changing environmental conditions is at the heart of research on evolutionary rescue.

Allelic simulation models, as used in this study, provide an ideal tool for integrating the available knowledge on eco-evolutionary dynamics from different organizational levels and to reflect the complex nature of adaptive and demographic processes. However, to date, most modelling studies have been highly abstracted, for example, assuming unrealistically high mutation rates and panmictic populations. Here, we have taken a first step towards quantitative predictions of population response to environmental change by establishing an individual-based model that is both spatially and genetically explicit, and that, as far as possible, has been parametrized realistically for both genetic and demographic functions.

The initial results of our model presented within this paper demonstrate two potent key phenomena that we consider important, particularly under ongoing habitat deterioration and fragmentation: first, the potentially complex effects of dispersal for a population's evolutionary response to both spatially heterogeneous habitats and shifting climate. And second, the possibility for partial evolutionary rescue, whereby rapid adaptation saves a population from extinction, but both population size and its geographical range may be substantially reduced.

Considering the effects of dispersal on local adaptation and environmental change separately, the results of our model concur with existing studies on each topic. Under habitat heterogeneity and local adaptation, dispersal typically has negative consequences for the average fitness [36,37]. Increased migration load—in our model output reflected by reduced levels of adaptation to the local environment—lead to higher mortality rates and an increased risk of location extinction, hence a lower chance of rescue. On the other hand, as argued and shown recently by Bell & Gonzalez [35], greater dispersal can be strongly beneficial, owing to its function in spreading favourable alleles across the populations' distributional ranges. This was mirrored by our results, when focusing on only the adaptation to temporally changing climate and thus neglecting the distorting effects of migration load.

The interplay of these double-edged consequences of gene flow leads to the key results that we emphasize in this paper. When dispersal is high and habitat heterogeneous, the number of viable offspring in each generation can be drastically reduced due to the arrival of many maladapted juveniles. At the population level, this is of little consequence when the climate is stable, as long as the number of surviving juveniles can maintain the population in a steady state. However, when the population needs to adapt to new climatic conditions, the absolute number of beneficial mutations becomes crucial. This number depends not only on the mutation rate, but also on the number of potential recruits that may carry these mutations and pass them on to subsequent generations. High rates of juvenile dispersal into habitat to which they are ill-adapted reduces the effective rate at which beneficial mutations on climate-related loci can be fixed in the population (see Barton & Bengtsson [52]). Ultimately, this interaction between dispersal, habitat heterogeneity and temporal environmental change leads to the observed reduction in the probability of evolutionary rescue. This suggest that even under high dispersal scenarios, populations previously adapted to spatially structured local environments may have a lower chance to adapt to changing regional climate.

The second key result—partial evolutionary rescue—is in its mechanism closely linked to the process described above. High habitat heterogeneity, subsequent migration load and decreased survival probability hamper the spatial spread of beneficial alleles, which may become locally abundant. The positive fitness effect of the beneficial mutation on climate-related loci becomes overridden by the negative effects due to genetic swamping by newly arrived individuals carrying alleles that are not adapted to local environmental conditions. This is obviously most likely when habitat is strongly heterogeneous. Thus, when the resulting absolute fitness of these individuals is lower than unity, beneficial alleles cannot spread throughout the distributional range of the population, thus preventing a species fully recovering its original geographical range following a shift in regional climate. In case the surviving subpopulations are too small to supply a sufficient amount of new mutations for adaptation to the conditions in the unpopulated space, we tend to observe a quasi-stable fragmented distribution of the surviving populations.

Our model also demonstrates that different ecological traits—even though not genetically correlated—may interact with the evolutionary dynamics, because they have additional effects on individuals' fitnesses and ultimately on populations' demographic rates. It seems that linkage disequilibrium between adaptive loci indeed has a prominent effect on the chance of evolutionary rescue. We found evolutionary rescue to be more likely under total genetic independence than under full linkage between adaptive loci. These results are not straightforward given our model structure. First, we could have expected that under low linkage between adaptive loci, the evolutionary response to shifting regional climate could be reduced, because stabilizing selection for local environments would account for most genetic load (i.e. for most fitness reduction). Second, one could also expect that adaptive response to changing climate would be reduced when recombination between climate-related loci can occur at every generation, thus breaking apart adaptive allele combinations and preventing the population from being fully rescued. Whether and how linkage may facilitate or impede adaptation to changing environmental conditions could be further investigated with our model, but is beyond the scope of this paper.

Clearly, a number of genetic, demographic and environmental settings that were neglected in this study can modulate the effects of spatio-temporal variability on micro-evolutionary dynamics. Some of these are shortly discussed in the following.

In terms of the genetic basis of adaptation, it has been shown that the relative amount of genetic versus environmental variability in individual phenotypes affects the speed of adaptation and the likelihood of evolutionary rescue [39,53]. While the probability of population extinction is increased under lower heritability of those traits controlling adaptation to temporally changing conditions, for traits controlling adaptation to spatial heterogeneity, low heritabilities and high plasticity may instead facilitate population survival: plasticity can buffer the negative effects of local maladaptation, reduce mortality and thus allow for increased effective dispersal and the spread of beneficial alleles. Weaker selection will have a positive influence on the survival probability of populations as well, because the effects of maladaptation are reduced. This effect is more pronounced when the habitat is heterogeneous (see the electronic supplementary material, figure S6), because the level of adaptation to both climate and local conditions determine population development in this case. Furthermore, a number of studies have demonstrated that characteristics of allelic effects such as epistatis or pleiotropy [54] and the nature of the selection (i.e. hard versus soft selection) [55] might change evolutionary dynamics substantially.

Focusing on demographic effects on rapid adaptation, the characteristics and effects of dispersal and gene flow may need more detailed inspection. For example, gene flow by pollen will affect adaptation processes differently compared with gene flow by dispersal of seeds or individuals [37]. First, the expected level of migration load is only half as high for pollen as for seed dispersal, because just half the number of maladapted alleles are placed into a new local environment, leading to decreased mortality. Second, the direct effect of shifting individuals between locations does not apply, partly decoupling evolutionary from demographic dynamics. Apart from that, it has to be considered that dispersal capabilities evolve rapidly themselves [56–58]. This adds another layer of complexity to forecasting population dynamics in space and time, but should generally increase populations’ survival probabilities. Furthermore, the tree types of population response—plasticity, adaptation and migration—are not mutually exclusive. Whenever populations are not limited in their distribution and tracking of suitable habitat is possible, the balance between positive and negative effects of dispersal has to be reconsidered.

Finally, in the context of environmental conditions, it should be noted that particularly when habitat is heterogeneous, the condition changing temporally may show variability across space. In this case, contrary to its effect demonstrated in this study, spatial heterogeneity may even accelerate adaptation to temporal change by increasing the genetic variance on which evolution can operate [42,59].

## 5. Conclusion

In past years, some remarkable studies have been published identifying the genetic basis for variation in traits that are important for adaptation under climate change [60–64]. If we are to understand under which conditions species will be able to build upon this variation to respond to environmental change, an important next step is now to scale up the knowledge of the genetics underpinning adaptation to the level of population demography. In a recent study, Chevin *et al.* [5] present a relatively simple evolutionary model to assess—for a given combination of phenotypic variance, heritability, selection strength, growth rate and plasticity—the critical rate of environmental change beyond which a population must decline and go extinct. This type of analytical model allows for a rigid mathematical analysis and can give valuable insights into the sensitivity and interdependence of parameters. On the other hand, many of the typically complex dynamics of evolutionary processes in natural populations cannot be captured. Thus, we believe that the type of allelic simulation model we applied in our study will be needed, if we are to ultimately make robust quantitative predictions on the likelihood of evolutionary rescue in particular populations or species. Here, we could show that the evolutionary potential of populations facing deteriorating conditions might be overestimated when neglecting the effects of local adaptation to heterogeneous habitat characteristics. This finding will be important, because increasing habitat deterioration will lead to reduced total habitat availability, increased habitat fragmentation and stronger spatial habitat heterogeneity, all of which are likely to impede the ability of species to track their preferred climate.
